# Progress in State Medicine

**Published:** 1900-02-03

**Authors:** 


### PROGRESS IN STATE MEDICINE.

### VITAL STATISTICS.

**Statistics of Population.**—The population of England and Wales[1] in 1570 has been calculated at about 4,160,321. In 1891 it reached 29,081,962. Upon the commencement of the nineteenth century the percentage increase of population did not average in 50 years more than 10 per cent., and in 1801 the populati on was only 8,872,980. The enormous increase in the last 90 years is due chiefly to the growth of manufacturing industries, but is also partly due to a great diminution of mortality, and an increased birth-rate, The growth of national prosperity and the cheapening of food have greatly assisted in this rapid rise of population. Whilst the exact figures relating to emigration and immigration are not known; it is nevertheless certain that a greater number of persons leave this country than enter it from without. Population is increasing most rapidly in England, Germany, and Russia; but in America it has multiplied itself 21 times in 110 years. There are many points showing that food production increases even more rapidly than population. For instance, there is as yet no return of urban population to agricultural pursuits; the number of paupers is decreasing; tea, tobacco, and other luxuries are more widely consumed than ever; bread is cheaper; wages are higher. The stationary population of France, and the diminishing birth-rates of England and America are due to the employment of means to prevent conception. If, however, the rate of emigration to the United States were to continue as at present the density of population would be equal to that of England in a hundred years. There are at present no grounds for fearing over-population. There is need for a very great improvement in our methods of census-taking. The chief desiderata are[2]: (1) It must be an accurate and complete statement. This entails skilled enumerators. (2) The census must be a quinquennial one. This will lead to accurate vital statistics at intercensus periods. The cost of a census is about £5 5s. per 1,000. (3) The enumerators' districts should be arranged in concert with the medical officers of health, and the sanitary districts assimilated to the registration districts. (4) The exact age and sex of each person must be ascertained. (5) The social condition of the people should be more closely inquired into. Thus, the distinction between those who produce and those who distribute, between employer and employed, and between makers and dealers, should be brought out. A greater thoroughness with regard to the occupation of tenement houses is to be hoped for.

Willoughby[3] mentions the following as being amongst the most frequent mistakes arising from the abuse of statistics: (1) Errors based on the assumption that the rate of increase observed in the last decennium is maintained in the present. Thus at the 1891 census the population of London was found to be a quarter of a million less than the estimate. (2) Errors in calculating the age and sex constitution of a population will alter the standard death-rate. (3) Reckoning the general longevity by the proportion which the number of old people bears to the total population. The natural increase of a population depends not only on the number and fertility of the marriages but on the age at which girls are married. The fertility of women is to be calculated not on the number of marriages, but of women who have married, in this way allowing for the remarriage of widows. In dealing with the comparative healthiness of occupations it is necessary to compare the death-rate per thousand living with that of the general population of the same age and sex. In all cases the possibility of a plurality of causes should not be lost sight of. The only legitimate comparisons are between those in which all conditions, save the one in question, are practically identical.

Some interesting figures with regard to the comparative mortality of English districts are given by Parsons.[4] The annual death-rate in England and Wales from 1847-57 was 23·1, from 1892-96 it was 18·1. At the present time there are very wide variations in the death-rates of our large towns; thus from 1888-97 the death-rate of Croydon was 14·4, of Liverpool 25·5. Similarly, comparing town and county, we find that the rate for the 33 large towns was 19, for the 67 towns of the next size 17·2, and in the rest of England and Wales 16·3. The difference in mortality in town and county districts is diminishing. The higher mortality amongst town dwellers is due to a higher infantile mortality and a higher zymotic rate. Comparing county areas we find variations from 14·8 in Surrey to 21·8 in Lancashire. Drawing a line from the mouth of the Severn to the Wash, the unhealthy counties will all lie to the north. Measles, scarlet fever, whooping-cough, and diarrhœa have higher rates in the urban, manufacturing, and mining areas than in agricultural counties. This is also true of enteric fever, the mortality being highest in Durham and Lancashire, lowest in Wiltshire and Oxfordshire. Speaking generally, enteric fever is most fatal where the midden privy system is in vogue. Diphtheria is now higher in town than in country. London and Essex have the highest rates. Influenza is below the average in the manufacturing and mining areas, above the average in the southern and midland agricultural counties, and in the hilly counties towards the west coast. Phthisis is fairly evenly distributed, but the death-rate varies from 1·08 per 1,000 in Worcestershire to 1·89 in Northumberland. The death-rate from diseases of the respiratory organs is low in the agricultural counties; high in the large smoky towns, where the population is engaged in dusty occupations. The hilly counties of the West, Gloucester, Hereford, Cumberland, Westmoreland, have the highest rates from diseases of the heart and circulatory system. The higher rate of mortality in the northern towns is dependent upon defective housing, the midden-privy system, the industrial occupations, and the presence of a strong Celtic element. These factors are of more importance than overcrowding of tenements or than density of population. Tatham[5] finds the death-rate of occupied males between the ages of 25 and 65 to be much lower than that of the unoccupied. This is accounted for by the fact that the latter class is a small one, and includes the inhabitants of workhouses, asylums, hospitals, &c.

The comparative mortality figure for all males was 953; it was 687 in the agricultural districts, and 1,248 in industrial districts, this wide difference being in great part due to housing, feeding, and sanitation. The lowest death-rate is that of the clergy, 533; whilst that of the schoolmaster (604) comes next. The comparative rate in the legal profession is 821, and in the medical 966. Farmers and gardeners show a very low mortality, and the rate for shopkeepers was only 859. All occupations connected with the liquor traffic show high rates—brewers, 1,427; publicans, 1,642; hotel servants, 1,725. High rates of mortality were constant amongst cutlers, file makers, nail makers, slaters, tilers, wool, silk, and cotton dyers, potters, and glass manufacturers. Taking the standard as 100, we find that the comparative figures amongst publicans and other servants are 723 for alcoholism, 600 from gout, 271 from diabetes, 644 from diseases of the liver, 210 from urinary diseases, and 207 from suicide. The injurious effects of dust-laden air are shown by the fact that the mortality from phthisis amongst file makers, cutlers, potters, and earthenware manufacturers is four times greater than that amongst the general public.

The following estimates have been made with regard to the amount of puerperal mortality.[6] Coghlan gives the rate as 1 in 142, McClintock 1 in 126, Matthews Duncan 1 in 120, Boxall 1 in 200, Registrar-General 1 in 200. Anderson regards these figures as too high, and from an examination of 71,122 deliveries fixes the rate as 1 in 563·6. These last figures being taken from outdoor maternities cannot, however, be accepted, as frequently when such cases go badly the charity is deserted and outside help brought in. Wallace and Playfair, from an analysis of the cases at the Edinburgh Royal Maternity and Simpson Memorial Hospital, fix the rate at 1 in 115. Amongst the difficult cases brought into the hospital the rate was 1 in 62, whilst the mortality figure amongst patients treated at home was 1 in 194.

**Chemical Preservatives in Food.**—The Local Government Board is now conducting an inquiry into this matter. Evidence is given by medical and chemical experts and by members of the provision trade. Borax would appear to be the preservative most widely used; 75 per cent. of foreign bacon is thus preserved, the average quantity used being 1 per cent. Much of this, however, is washed off before sale. Evidence was given showing that 50 per cent. of milk samples taken in London showed preservatives, boracic and formalin figuring about equally. Butter was generally preserved with borax, jam with salicylic acid (1 part in 3,500), cider and home wines with salicylic acid, and meat peptones, beef jellies, and other sick-room articles of diet by boracic acid. Annato is used for colouring milk and butter, magenta (1 in 100,000) and vermilion (1 in 80,000) for jams, cochineal for preserved cherries, and sulphate of copper (from 2 to 3 grains per pound) for peas, beans, and spinach. Raisins, apricots, and plums are bleached by sulphur. Evidence was given by the Medical Officers of Health of Glamorgan County, Notts County, Birmingham, and Leeds showing the widespread nature of the adulteration. The last three witnesses were agreed that serious digestive disturbance and ill-health might follow the continued ingestion of such preservatives. Dr. Wild, on the other hand, did not think that bad consequences were likely to arise from the quantities now used in foods. On this point there is much diversity of opinion. Certainly few authenticated cases of poisoning are on record. Robinson[7] gives details of a family of five persons poisoned by eating a blanche mange containing a large quantity of borax and Annett,[8] who fed kittens on milk containing either borax (40-80 grains per gallon) or formalin (1 in 25,000), found that they either lost weight, died, or failed to develop as well as control animals. Neither of these instances are quite satisfactory, and the conclusions drawn from their observations are strongly combated by Liebreich,[9] who points out that in the former instance ptomain or metallic poisons were not excluded, and in the latter large doses of the drugs were administered, such as we knew to be toxic. Test tube experiments show that formalin, boric acid, and more especially borax, have in large quantities an inhibiting and retarding action on the digestion of starches and proteids, but this action is negligable when we are dealing with the amounts necessary for food preservation, *e.g.*, milk with 35 grains of boric acid per gallon, or formalin to the extent of one part in 50,000. Foulerton, who, with Rideal,[10] has done much work on this subject, points out that the danger is of course greater from the fact that the largest consumers of milk are young children and invalids. Benzoic acid, which is free from many of the obvious objections applying to other preservatives, is but little used. Seventeen grains per gallon is sufficient to preserve milk.

[1] Practitioner, Sept., 1899. [2] Ibid, Oct., 1899. [3] Jour. San. Inst., April, 1899. [4] Lancet, Nov. 25, 1899. [5] Report on Occupational Mortality. [6] Brit. Med. Jour., Sept. 17, 1898. [7] Public Health, Aug., 1899. [8] Lancet, Nov. 11, 1899. [9] Ibid, Jan. 6, 1900. [10] Public Health, Vol. xi., No. 3, 1899.

